# Multimodal Physical Therapy Management of Subcoracoid Impingement: A Case Report With One-Year Follow-Up and Ultrasound Measurement of Coracohumeral Distance

**DOI:** 10.7759/cureus.73398

**Published:** 2024-11-10

**Authors:** Abdallah Gamiel, Hosny Elkhawaga, Mohamed Badr, Yousef M Abdullatif, Mohamed Amr

**Affiliations:** 1 Department of Physical Therapy for Musculoskeletal Disorders and Its Surgeries, Faculty of Physical Therapy, Modern University for Technology and Information (MTI), Cairo, EGY; 2 Faculty of Physical Therapy, Cairo University, Giza, EGY; 3 Department of Physical Therapy for Neuromuscular Disorders and Its Surgery, Faculty of Physical Therapy, Al Hayah University in Cairo, Cairo, EGY; 4 Department of Biomechanics, Faculty of Physical Therapy, Modern University for Technology and Information (MTI), Cairo, EGY; 5 Department of Orthopedic Surgery, Faculty of Medicine, Ain Shams University, Cairo, EGY

**Keywords:** coracoid impingement, diagnostic musculoskeletal ultrasound, orthopaedics and sports physical therapy, physical therapy, physical therapy rehabilitation, physiotherapy intervention, shoulder impingement, sports physiotherapy, sports ultrasound, subcoracoid impingement

## Abstract

Subcoracoid impingement occurs due to mechanical encroachment of the subscapularis tendon in the subcoracoid space between the coracoid process and lesser tuberosity of the humerus. Although physical therapy is known to have a crucial role in managing this condition, to the best of our knowledge, there is no established physical therapy program in the literature. This case report aims to provide a detailed presentation and diagnosis of a subcoracoid impingement case and to investigate the effects of physical therapy on pain, disability, performance, muscle strength, and ultrasound measurements over a one-year follow-up period. The patient was a 24-year-old male working as a jeweler who had been suffering from dull anterior left shoulder pain for five years. The modified Hawkins-Kennedy test was positive. Additionally, palpation was pain-free, except for severe pain in the coracoid area. The patient was injected with xylocaine into the subcoracoid space and demonstrated a spontaneous relief of pain. Ultrasound imaging showed a narrower coracohumeral distance from full internal rotation on the affected side (0.85 cm) compared to the non-affected side (1.22 cm). Six weeks of multimodal physical therapy program was delivered to the patient. It consisted of electrophysical agents, manual therapy, and therapeutic exercise. Electrophysical agents included conventional transcutaneous electrical nerve stimulation, ice, and phonophoresis. Manual therapy included shoulder mobilization, myofascial release, thoracic mobilization, and posterior capsule stretches. Additionally, scapular muscle-strengthening and Rotator cuff strengthening exercises were delivered to the patient. The patient received 18 sessions for 6 weeks, at a rate of three times per week. Shoulder pain, function, and performance were measured by a numeric rating pain scale, shoulder pain and disability index, and timed push-up test, respectively. The shoulder muscle's peak isometric strength was measured by a hand-held dynamometer. Acromiohumeral distance, coracohumeral distance, supraspinatus thickness, and subscapularis thickness were measured by ultrasound imaging. Six weeks of multimodal physical therapy is a successful intervention for patients with subcoracoid impingement. It resulted in improvements in pain, function, performance, and muscle strength. An increase in coracohumeral distance from full internal rotation was observed at the end of the intervention, as well as after three months and one year.

## Introduction

Shoulder impingement is one of the most common musculoskeletal conditions in general practice [1]. It occurs due to mechanical encroachment of the rotator cuff tendons, which leads to inflammation and irritation [1]. Subcoracoid impingement is a type of shoulder impingement that is relatively uncommon compared to subacromial impingement [2]. While subacromial impingement occurs due to mechanical encroachment of the rotator cuff in the subacromial space [3], subcoracoid impingement occurs due to mechanical encroachment of the subscapularis tendon in the subcoracoid space between the coracoid process and lesser tuberosity of the humerus [4]. The etiology of subcoracoid impingement may include idiopathic factors such as anatomical variations of the coracoid process, ganglion cysts, and ossification or calcification of the subscapularis tendon. Additionally, iatrogenic factors from surgical procedures and traumatic factors related to anterior instability may also contribute to the condition [5].

Diagnosing subcoracoid impingement can be challenging and requires a combination of patient history, clinical examination, diagnostic injection, and appropriate imaging studies [5,6]. Regarding patient history, patients complain of dull anterior shoulder pain that is aggravated by forward flexion, adduction, and internal rotation of the shoulder and overhead activities. Clinical examination demonstrates limited range of motion in horizontal adduction/internal rotation, tenderness with palpation around the coracoid process, and positive coracoid impingement test, which is performed as Kennedy-Hawkins test, except that the shoulder is placed in a position of cross-arm adduction to bring the lesser tuberosity in contact with the coracoid. Additionally, relief of pain with injection of xylocaine into the subcoracoid region can help to establish the diagnosis [4]. Direct arthroscopic observation of the subcoracoid contact is the final way to make the diagnosis [7]. However, several imaging studies including X-ray, computed tomography (CT) scan, magnetic resonance imaging (MRI), and ultrasound help in diagnosis [5,6]. Ultrasonography allows dynamic real-time evaluation of the subcoracoid region and can be used for diagnosing coracoid impingement [8]. Tracy et al. [9] found that coracohumeral distance was narrower in patients with clinically diagnosed coracoid impingement (7.9 ± 1.4 mm) compared to healthy volunteers (12.2 ± 2.5 mm).

Management of subcoracoid impingement initially involves conservative treatments, including rest, anti-inflammatory medications, and physical therapy. If these conservative treatments fail, surgical intervention may be considered [6]. Although physical therapy is known to have a crucial role in managing this condition, to the best of our knowledge, there is no established physical therapy program in the literature. This case report aims to provide a detailed presentation and diagnosis of a subcoracoid impingement case and to investigate the effects of physical therapy on pain, disability, performance, muscle strength, and ultrasound measurements over a one-year follow-up period.

## Case presentation

Patient history

This study was conducted from May 2023 to May 2024. An informed consent was obtained from the patient. The patient was a 24-year-old male working as a jeweler who had been suffering from dull anterior left shoulder pain for five years (since 2018). He reported high irritability with the pain, rating it 9 out of 10 on the Numeric Rating Pain Scale (NRPS), accompanied by a sense of tightness in the posterior aspect of the left side of his neck. The pain was aggravated by external rotation, and his chief complaint was the inability to lift heavy objects with his left shoulder.

This problem affected his work and his ability to go to the gym. As a jeweler, he often sits in a slouched position for long periods. The pain began gradually in 2018 and has since intensified, preventing him from going to the gym. He underwent subacromial corticosteroid injections in his shoulder twice, in 2019 and 2020, but did not experience any relief.

Clinical examination

By observation, scapular dyskinesis pattern II was noted on the left shoulder [10]. Cervical spine pathology was excluded radiologically by clear MRI. Additionally, a clinical examination of the cervical spine revealed intact sensory, motor, and reflex responses, with negative findings for the spurling, distraction, upper limb tension test 1, and shoulder abduction tests [11]. Moreover, thoracic outlet syndrome was excluded based on negative Adson's and Roos tests [12]. Subacromial impingement was ruled out by negative Neer, Kennedy-Hawkins, painful arc, and empty can tests [13]. The Biceps Load II test was positive, while the Speed test was negative [14]. Range of motion assessment revealed a full range of motions with no pain, except during active external rotation, which was painful. 

Regarding the subcoracoid examination, the modified Kennedy-Hawkins test was positive. This test was performed similarly to the Kennedy-Hawkins test, with the shoulder placed in a position of cross-arm adduction. Additionally, palpation was pain-free, except for severe pain in the coracoid area [5,6].

Diagnostic injection and ultrasound imaging

The patient was injected with xylocaine into the subcoracoid space and demonstrated a spontaneous relief of pain, which increased the probability of the diagnosis of subcoracoid impingement [4]. Ultrasound imaging showed a narrower coracohumeral distance from full internal rotation on the affected side (0.85 cm) compared to the non-affected side (1.22 cm). Regarding the subacromial space, the acromiohumeral distance was comparable on both sides, which rules out subacromial impingement.

Multimodal physical therapy intervention

Six weeks of multimodal physical therapy program was delivered to the patient. It consisted of electrophysical agents, manual therapy, and therapeutic exercise. The patient received 18 sessions for 6 weeks, at a rate of three times per week.

Electrophysical Agents

Conventional transcutaneous electrical nerve stimulation (TENS), ice, and phonophoresis were delivered to the patient. From the supine position, one electrode was placed on the anterior shoulder and the other on the lateral aspect of the shoulder along with an ice pack over the shoulder region for 20 min. Low-intensity pulsed ultrasound phonophoresis with diclofenac and dexamethasone was administered on the coracoid area for 5 minutes [15].

Manual Therapy

Shoulder posterior glide and inferior glide mobilization [16], Myofascial release for pectoralis major and minor, subscapularis, latissimus dorsi, levator scapulae, and upper trapezius [17], thoracic mobilization [18], and posterior capsule stretch [19] were delivered to the patient.

Therapeutic Exercises

Scapular muscle-strengthening and Rotator cuff strengthening exercises were delivered to the patient. Scapular muscle-strengthening exercises included shoulder extension with theraband from supine and standing [20], seated row resistance exercise [21], prone extension, and prone horizontal abduction with external rotation [22,23]. Rotator cuff strengthening exercises included external and internal rotation and scaption with weight exercises [20,24]. External and internal rotation exercises were performed from two positions: standing using a theraband with a pillow under the elbow, and at 90 degrees of abduction and external rotation [24]. Each exercise was performed in three sets of 10 repetitions.

After six weeks of multimodal physical therapy, the patient returned to the gym and was instructed to continue with scapular muscle-strengthening and rotator cuff strengthening exercises. The detailed multimodal physical therapy program in each session is shown in Table 1.

**Table 1:** Detailed multimodal physical therapy program in each session Abbreviations: TENS; transcutaneous electrical nerve stimulation

		Electrophysical agents	Manual Therapy	Therapeutic Exercises
Week 1	Session 1	Conventional TENS, ice, and phonophoresis	Thoracic mobilization	Shoulder extension with theraband from supine and standing
	Session 2	Conventional TENS, ice, and phonophoresis	Thoracic mobilization	Shoulder extension with theraband from supine and standing
	Session 3	Conventional TENS, ice, and phonophoresis	Thoracic mobilization	Shoulder extension with theraband from supine and standing
Week 2	Session 4	Conventional TENS, ice, and phonophoresis	Shoulder posterior glide and inferior glide mobilization, Myofascial release for pectoralis major and minor, subscapularis, latissimus dorsi, levator scapulae, and upper trapezius, and thoracic mobilization	Shoulder extension with theraband from supine and standing, prone extension, and external rotation exercises from standing using a theraband with a pillow under the elbow.
	Session 5	Conventional TENS, ice, and phonophoresis	Shoulder posterior glide and inferior glide mobilization, Myofascial release for pectoralis major and minor, subscapularis, latissimus dorsi, levator scapulae, and upper trapezius, and thoracic mobilization	Shoulder extension with theraband from supine and standing, prone extension, prone horizontal abduction with external rotation, and external rotation exercises from standing using a theraband with a pillow under the elbow.
	Session 6	Conventional TENS, ice, and phonophoresis	Shoulder posterior glide and inferior glide mobilization, Myofascial release for pectoralis major and minor, subscapularis, latissimus dorsi, levator scapulae, and upper trapezius, and thoracic mobilization	Shoulder extension with theraband from supine and standing, prone extension, prone horizontal abduction with external rotation, and external rotation exercises from standing using a theraband with a pillow under the elbow.
Week 3	Session 7	Conventional TENS, ice, and phonophoresis	Shoulder posterior glide and inferior glide mobilization, Myofascial release for pectoralis major and minor, subscapularis, latissimus dorsi, levator scapulae, and upper trapezius, and thoracic mobilization.	Shoulder extension with theraband from supine and standing, prone extension, prone horizontal abduction with external rotation, and external rotation exercises from standing using a theraband with a pillow under the elbow.
	Session 8	Conventional TENS, ice, and phonophoresis	Shoulder posterior glide and inferior glide mobilization, Myofascial release for pectoralis major and minor, subscapularis, latissimus dorsi, levator scapulae, and upper trapezius, and thoracic mobilization.	Shoulder extension with theraband from supine and standing, prone extension, prone horizontal abduction with external rotation, and external rotation exercises from standing using a theraband with a pillow under the elbow.
	Session 9	Conventional TENS, ice, and phonophoresis	Shoulder posterior glide and inferior glide mobilization, Myofascial release for pectoralis major and minor, subscapularis, latissimus dorsi, levator scapulae, and upper trapezius, and thoracic mobilization.	Shoulder extension with theraband from supine and standing, prone extension, prone horizontal abduction with external rotation, external rotation exercises from standing using a theraband with a pillow under the elbow, and scaption with weight.
Week 4	Session 10	Conventional TENS, ice, and phonophoresis	Shoulder posterior glide and inferior glide mobilization, Myofascial release for pectoralis major and minor, subscapularis, latissimus dorsi, levator scapulae, and upper trapezius, and thoracic mobilization.	Shoulder extension with theraband from supine and standing, prone extension, prone horizontal abduction with external rotation, external rotation exercises from standing using a theraband with a pillow under the elbow, and scaption with weight.
	Session 11	Conventional TENS, ice, and phonophoresis	Shoulder posterior glide and inferior glide mobilization, Myofascial release for pectoralis major and minor, subscapularis, latissimus dorsi, levator scapulae, and upper trapezius, and thoracic mobilization.	Shoulder extension with theraband from supine and standing, prone extension, prone horizontal abduction with external rotation, external rotation exercises from standing using a theraband with a pillow under the elbow, and scaption with weight.
	Session 12	Conventional TENS, ice, and phonophoresis	Shoulder posterior glide and inferior glide mobilization, Myofascial release for pectoralis major and minor, subscapularis, latissimus dorsi, levator scapulae, and upper trapezius, and thoracic mobilization.	Shoulder extension with theraband from supine and standing, prone extension, prone horizontal abduction with external rotation, external exercises from standing using a theraband with a pillow under the elbow, and scaption with weight.
Week 5	Session 13		Shoulder posterior glide and inferior glide mobilization, Myofascial release for pectoralis major and minor, subscapularis, latissimus dorsi, levator scapulae, and upper trapezius, and thoracic mobilization.	Shoulder extension with theraband from supine and standing, seated row resistance exercise, prone extension, prone horizontal abduction with external rotation, external and internal rotation exercises from standing using a theraband with a pillow under the elbow , and scaption with weight.
	Session 14		Shoulder posterior glide and inferior glide mobilization, Myofascial release for pectoralis major and minor, subscapularis, latissimus dorsi, levator scapulae, and upper trapezius, and thoracic mobilization.	Shoulder extension with theraband from supine and standing, seated row resistance exercise, prone extension, prone horizontal abduction with external rotation, external and internal rotation exercises from standing using a theraband with a pillow under the elbow, and scaption with weight.
	Session 15		Shoulder posterior glide and inferior glide mobilization, Myofascial release for pectoralis major and minor, subscapularis, latissimus dorsi, levator scapulae, and upper trapezius, and thoracic mobilization.	Shoulder extension with theraband from supine and standing, seated row resistance exercise, prone extension, prone horizontal abduction with external rotation, external and internal rotation exercises from standing using a theraband with a pillow under the elbow, and scaption with weight.
Week 6	Session 16		Shoulder posterior glide and inferior glide mobilization, Myofascial release for pectoralis major and minor, subscapularis, latissimus dorsi, levator scapulae, and upper trapezius, thoracic mobilization, and posterior capsule stretch	Shoulder extension with theraband from supine and standing, seated row resistance exercise, prone extension, prone horizontal abduction with external rotation, external and internal rotation exercises from standing using a theraband with a pillow under the elbow and at 90 degrees of abduction and external rotation , and scaption with weight.
	Session 17		Shoulder posterior glide and inferior glide mobilization, Myofascial release for pectoralis major and minor, subscapularis, latissimus dorsi, levator scapulae, and upper trapezius, thoracic mobilization, and posterior capsule stretch	Shoulder extension with theraband from supine and standing, seated row resistance exercise, prone extension, prone horizontal abduction with external rotation, external and internal rotation exercises from standing using a theraband with a pillow under the elbow and at 90 degrees of abduction and external rotation , and scaption with weight.
	Session 18		Shoulder posterior glide and inferior glide mobilization, Myofascial release for pectoralis major and minor, subscapularis, latissimus dorsi, levator scapulae, and upper trapezius, thoracic mobilization, and posterior capsule stretch	Shoulder extension with theraband from supine and standing, seated row resistance exercise, prone extension, prone horizontal abduction with external rotation, external and internal rotation exercises from standing using a theraband with a pillow under the elbow and at 90 degrees of abduction and external rotation, and scaption with weight.

Outcomes

Shoulder pain, function, and performance were measured by NRPS, shoulder pain and disability index (SPADI) [25], and timed push-up test [26], respectively. The shoulder muscle's peak isometric strength was measured by a hand-held dynamometer [27]. Acromiohumeral distance, coracohumeral distance, supraspinatus thickness, and subscapularis thickness were measured by ultrasound imaging [28]. Changes in Outcome Measures are presented in Table 2.

**Table 2:** Changes in Outcome Measures Abbreviations: NRPS: numeric pain rating scale, SPADI: shoulder pain and disability index

Outcomes	Baseline	After 6 Weeks	After 3 Months	After 1 Year
NRPS (0-10)	9	2	2	3
SPADI (%)	49.23	7.69	6.92	6.92
Timed push-up test	8	13.5	13.5	13.5
Shoulder external rotators peak isometric strength of at 0° abduction (kg)				
Affected shoulder	5.5	7.3		
Non-affected shoulder	12.5	13.4		
Shoulder external rotators peak isometric strength of at 90-90 abduction and external rotation (kg)				
Affected shoulder	4.3	5		
Non-affected shoulder	8.6	8.7		
Shoulder internal rotators peak isometric strength of at 0° abduction (kg)				
Affected shoulder	13.2	15.7		
Non-affected shoulder	16.2	18.9		
Shoulder internal rotators peak isometric strength of at 90-90 abduction and external rotation (kg)				
Affected shoulder	11.8	11.5		
Non-affected shoulder	11.9	14.2		
Shoulder scaption peak isometric strength (kg)				
Affected shoulder	8.4	8.5		
Non-affected shoulder	13.5	14.4		
Acromiohumeral distance at 0° of scapular plane shoulder elevation (cm)				
Affected shoulder	1.1	1.1	1.14	1.17
Non-affected shoulder	1.16	1.16	1.14	1.23
Acromiohumeral distance at 60° of scapular plane shoulder elevation (cm)				
Affected shoulder	0.98	1.08	0.95	1.03
Non-affected shoulder	1.02	1.1	1.18	1.19
Supraspinatus thickness (cm)				
Affected shoulder	0.58	0.55	0.55	0.54
Non-affected shoulder	0.77	0.73	0.74	0.41
Coracohumeral distance from full external rotation (cm)				
Affected shoulder	1.14	1.14	1.32	1.34
Non-affected shoulder	1.44	1.41	1.31	1.56
Coracohumeral distance from full internal rotation (cm)				
Affected shoulder	0.85	1.12	1.23	1.01
Non-affected shoulder	1.22	1.19	1.11	1.28
Subscapularis thickness (cm)				
Affected shoulder	0.65	0.44	0.39	0.43
Non-affected shoulder	0.55	0.52	0.42	0.43

**Figure 1:**
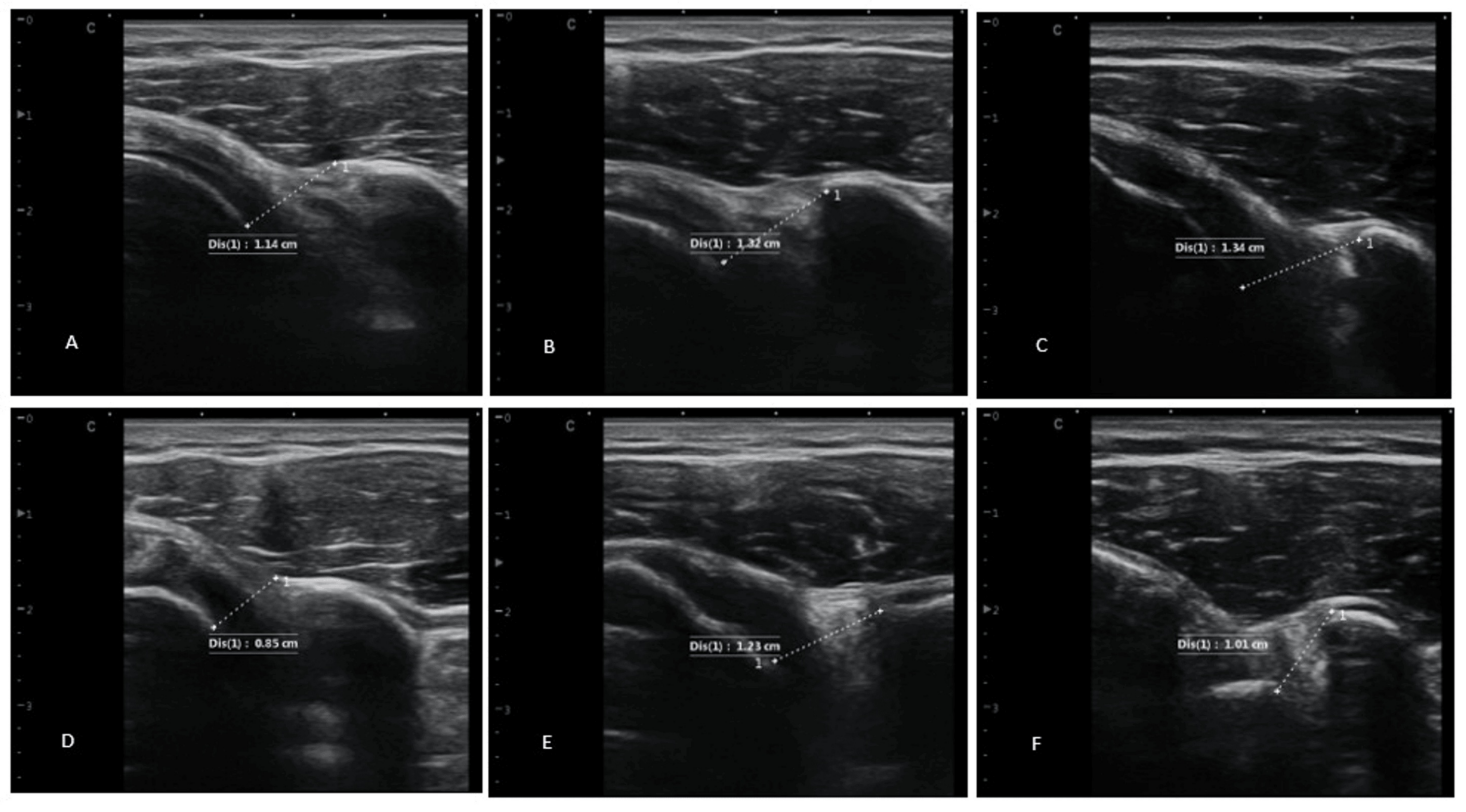
Coracohumeral Distance Measurements of the Affected Shoulder A) Coracohumeral distance from full external rotation at baseline. B) Coracohumeral distance from full external rotation after 3 months. C) Coracohumeral distance from full external rotation after 1 year. D) Coracohumeral distance from full internal rotation at baseline. E) Coracohumeral distance from full internal rotation after 3 months. F) Coracohumeral distance from full internal rotation after 1 year.

Coracohumeral distance measurements of the affected shoulder are shown in Figure 1.

## Discussion

This case report demonstrated that six weeks of multimodal physical therapy improved pain, function, performance, and muscle strength. Additionally, the coracohumeral distance from full internal rotation increased, and subscapularis thickness decreased, suggesting that multimodal physical therapy may positively affect biomechanics and tissue structure. Furthermore, improvements in pain, function, performance, coracohumeral distance, and subscapularis thickness were observed after three months and at one-year follow-up, supporting the long-term effectiveness of the physical therapy intervention.

The intervention in our study was progressive and multimodal, addressing all aspects of the condition. It included electrophysical agents, manual therapy, and therapeutic exercises to control pain, improve mobility and soft tissue restrictions, and increase neuromuscular control and strength. Conventional TENS, ice, and phonophoresis were delivered to modulate pain and prepare the patient for other interventions [15].

Shoulder mobilization [16], myofascial release [17], thoracic mobilization [18], and posterior capsule stretches were performed to improve glenohumeral joint mobility and the flexibility of the muscles and posterior capsule. Tightness in the posterior capsule can translate the head anteriorly and superiorly, which may decrease coracohumeral distance [29]. Therefore, posterior capsule stretches are important to be included with respect to patient irritability.

Scapular muscle-strengthening exercises were performed to increase scapular stability and improve scapular positioning in a retracted position, which may help increase coracohumeral distance [20-23]. Rotator cuff strengthening exercises were also conducted to enhance glenohumeral joint stability and maintain the humeral head's centralization in the glenoid, which may further increase coracohumeral distance [20, 24]. These exercises included internal rotation to load the subscapularis muscle. Notably, a decrease in subscapularis thickness was observed, which may be attributed to the increase in coracohumeral distance, reflecting a reduction in impingement mechanisms.

This case report has several strengths. To the best of our knowledge, it is the first to provide a detailed management approach for subcoracoid impingement from the physical therapy point of view. The patient’s diagnosis was confirmed through history, clinical examination, diagnostic injection, and ultrasound imaging. The physical therapy intervention was delivered in a multimodal form to address all aspects, including pain, mobility, flexibility, and stability. In addition to measuring pain, function, performance, and muscle strength, ultrasound was utilized to assess biomechanical changes. Additionally, follow-up assessments were conducted after three months and one year.

## Conclusions

Six weeks of multimodal physical therapy is a successful intervention for patients with subcoracoid impingement. It resulted in improvements in pain, function, performance, and muscle strength. An increase in coracohumeral distance from full internal rotation was observed at the end of the intervention, as well as after three months and one year.
